# Effect of Thoracic Squeezing Technique and Expiratory Flow Increase Technique on Neonates With Neonatal Respiratory Distress Syndrome: A Case Report

**DOI:** 10.7759/cureus.68702

**Published:** 2024-09-05

**Authors:** Samruddhi Aherrao, H V Sharath

**Affiliations:** 1 Department of Cardiorespiratory Physiotherapy, Ravi Nair Physiotherapy College, Datta Meghe Institute of Higher Education & Research (DU), Wardha, IND; 2 Department of Paediatric Physiotherapy, Ravi Nair Physiotherapy College, Datta Meghe Institute of Higher Education & Research (DU), Wardha, IND

**Keywords:** expiratory flow increase technique, mechanical ventilation, neonatal respiratory distress syndrome, percussion, reflexes

## Abstract

Neonatal respiratory distress syndrome (NRDS) is a major cause of morbidity and death in premature newborns due to inadequate surfactant synthesis in the lungs. Preterm birth carries a higher risk of respiratory problems. Clinically cyanosis, grunting, retractions, and tachypnea are signs of early respiratory distress associated with RDS Should therapeutic measures not be implemented, the infant may suffer respiratory failure and increasing hypoxia. This case study investigates how physical therapy affects a preterm newborn with NRDS regarding thoracic squeeze, percussion, and vibration with expiratory flow increase technique The patient’s prenatal, natal, and postnatal history is included in depth in the report, along with information on the mother's health, pregnancy difficulties, birth details, first clinical presentation, and care that followed. Despite advancements in treatment through the use of surfactants, prenatal corticosteroids, and advanced respiratory care for the newborn, RDS continues to be the leading cause of morbidity and mortality in premature newborns. The significance of interdisciplinary methods in improving the care and prognosis of newborns with NRDS is shown by this instance.

## Introduction

Neonatal respiratory distress syndrome (NRDS), sometimes frequently referred to as hyaline membrane sickness, is a common and occasionally fatal illness that mostly affects premature infants. Insufficient production of pulmonary surfactant, which reduces lung surface tension and prevents alveoli from collapsing during expiration, is the cause of the condition [1]. Clinically, cyanosis, grunting, retractions, and tachypnea are signs of early respiratory distress associated with RDS. The infant may die from respiratory failure and increasing hypoxia if treatment is not received. RDS is caused by the lungs’ anatomical immaturity and alveolar surfactant deficiency [2]. Preterm babies now have a better chance of surviving and reuniting with relatives if they are born between 22 and 23 weeks of gestation. Initial life support is increasingly being provided to infants born at lower perceived levels of viability-which in certain countries is presently regarded as low as 22 weeks of gestation [3].

Inadequate surfactant is the cause of NRDS, particularly in the early phases of lung development. The juvenile lung’s compliance is reduced by insufficient surfactant because it raises the surface tension in the alveoli and small airways. To keep the alveolus from collapsing or filling with fluid, the atmospheric pressures at the air-fluid interface must be properly regulated [4]. When unwell neonates require medical attention, mechanical ventilation (MV) is frequently required, particularly if the kid is preterm. Even though MV is typically administered for a brief period, it could be needed for a longer period. Poor long-term respiratory and developmental outcomes as well as short-term issues have been linked to MV [5]. Additionally, it has been demonstrated that in newborns and early children who need artificial ventilation for scheduled heart surgery, the distribution of ventilation is altered. Forced breathing caused a change in ventilation distribution from the dependent to the non-dependent lung and enhanced ventilation inhomogeneity as compared to spontaneous breathing [6]. Preventive approaches can be used to plan early developmental interventions to improve the development of at-risk preterm newborns [7].

Preterm newborns’ neurological, digestive, and pulmonary systems positioning is essential. The prone position has several advantages over the supine position, including increased PaO_2_, increased rib cage contribution to tidal volume (Vt), improved thoracoabdominal synchronization, less obstructive apneas, a greater vulnerability to stress, increased sleep duration, and so on [8]. Research on adults has demonstrated that variations in the body cavity’s orientation with respect to gravity have an impact on the pressure-volume properties of the chest. The chest wall causes the diaphragm's dome to tilt toward gravity, which also raises the breathing muscles’ tonic activity and passive tension [9]. Borderline personality disorder (BPD) is one long-term effect of RDS. An element of the etiology of BPD is related to lung development stunting and inflammation. Surfactant is absent from the immature lung of the preterm newborn in addition to poor fluid clearance, weak adherence, and immature vascular development. These variables further obstruct the proper development of the pulmonary and alveolar vasculature and raise the risk of lung injury and inflammation [10].

Major concerns for newborns in NICUs include mortality and long-term morbidity; nonetheless, deaths of term neonates during NICU hospitalization are thought to be common, with documented incidences ranging from 8% to 15% [11]. In this case report, the physical rehabilitation program uses positioning, manual airway clearing, thoracic squeezing, and expiratory flow increase technique and therapy approaches to enhance overall physiological stability and respiratory function. Providing a detailed summary of the neonate’s antenatal, natal, and postnatal history; moreover, detailing the specific rehabilitative techniques employed and their impact on the neonate’s clinical outcomes.

## Case presentation

Patient information

As narrated by the mother, the baby was born 32+6 weeks gestation (preterm) via lower section cesarian section to 37-year-old Garvida 2 Para 1 and L 1 via IVF conception on January 11, 2024, at 10:23 AM. As per the condition of the baby, the mother complained that the baby was having difficulty breathing, decreased activity, and problems during feeding. Thus, the baby was shifted to the NICU. Auscultation of the chest revealed bilateral grunting breath sounds in line with the NRDS. On assessment, the chest was asymmetrical and there was a decrease in lung volume. No congenital anomalies are present. Head circumference is 32 cm and chest circumference is 34 cm. As per the procedure of the facility, the child quickly went on non-invasive breathing support with continuous positive airway pressure (CPAP) and administered exogenous surfactant treatment. All the primitive reflexes are taken and given in Table 1.

**Table 1:** Primitive reflexes

Sr. No	Primitive Reflexes	
1	Palmar	Present
2	Sucking	Absent
3	Rooting	Present
5	Plantar	Present

On examination

The mother’s consent and informed permission were obtained before the assessment on the same day evening. The baby was kept on CPAP Fio_2_-40, PEEP-6 cmH2O. Therefore, the baby was not maintaining saturation. He was kept on 2 L of oxygen. On January 13, investigations were done, such as chest X-ray and blood gas analysis. The appearance, pulse, grimace, activity, and respiration (APGAR) was done on January 13 and noted as 6/10. Consolidation was found on the chest X-ray, and surfactant was provided to the baby. On January 14, a physiotherapy call was noted for chest clearance. Thus, after the chest physiotherapy, there was an improvement in the condition of the baby and the recovery took place. Twice a day treatment was given. Respiratory parameters like heart rate, respiratory rate, and oxygen saturation were taken. After 2 weeks of chest physiotherapy treatment, the baby was in good in health and the prognosis was better. He was discharged on January 28 and the treatment was discontinued.

Investigation

**Figure 1:**
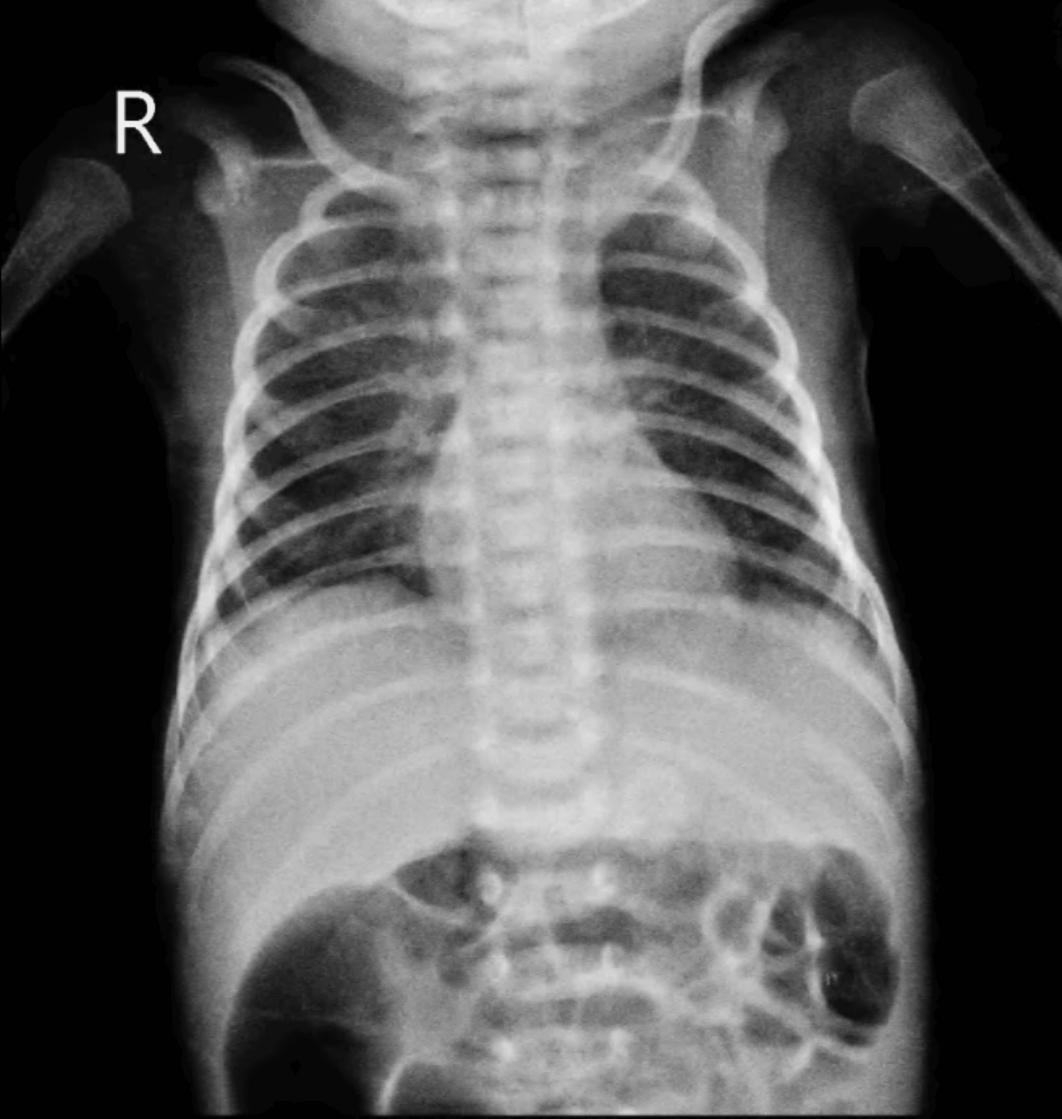
Consolidation present in left lower zones and bronchovesicular markings are present in the left upper, middle, and lower zones and right middle zones. Heterogenous opacity is present in the right middle and left lower zones.

The anteroposterior (AP) chest X-ray of a neonate with NRDS reveals consolidation in the left lower lung zones, characterized by increased opacity. The affected area displays homogeneous density, suggesting alveolar filling, which may be due to fluid accumulation, infection, or atelectasis. The heart and mediastinum are normal in size and position, with no evidence of mediastinal shift. The diaphragm is well-defined, although the left hemidiaphragm appears slightly elevated, potentially due to underlying lung pathology. The costophrenic angles are clear on the right but are partially obscured on the left due to the consolidation. No pleural effusion or pneumothorax is noted. The presence of consolidation in the left lower lung field is consistent with the clinical diagnosis of NRDS and should be evaluated alongside the neonate’s overall clinical condition mentioned in Figure 1.

**Figure 2:**
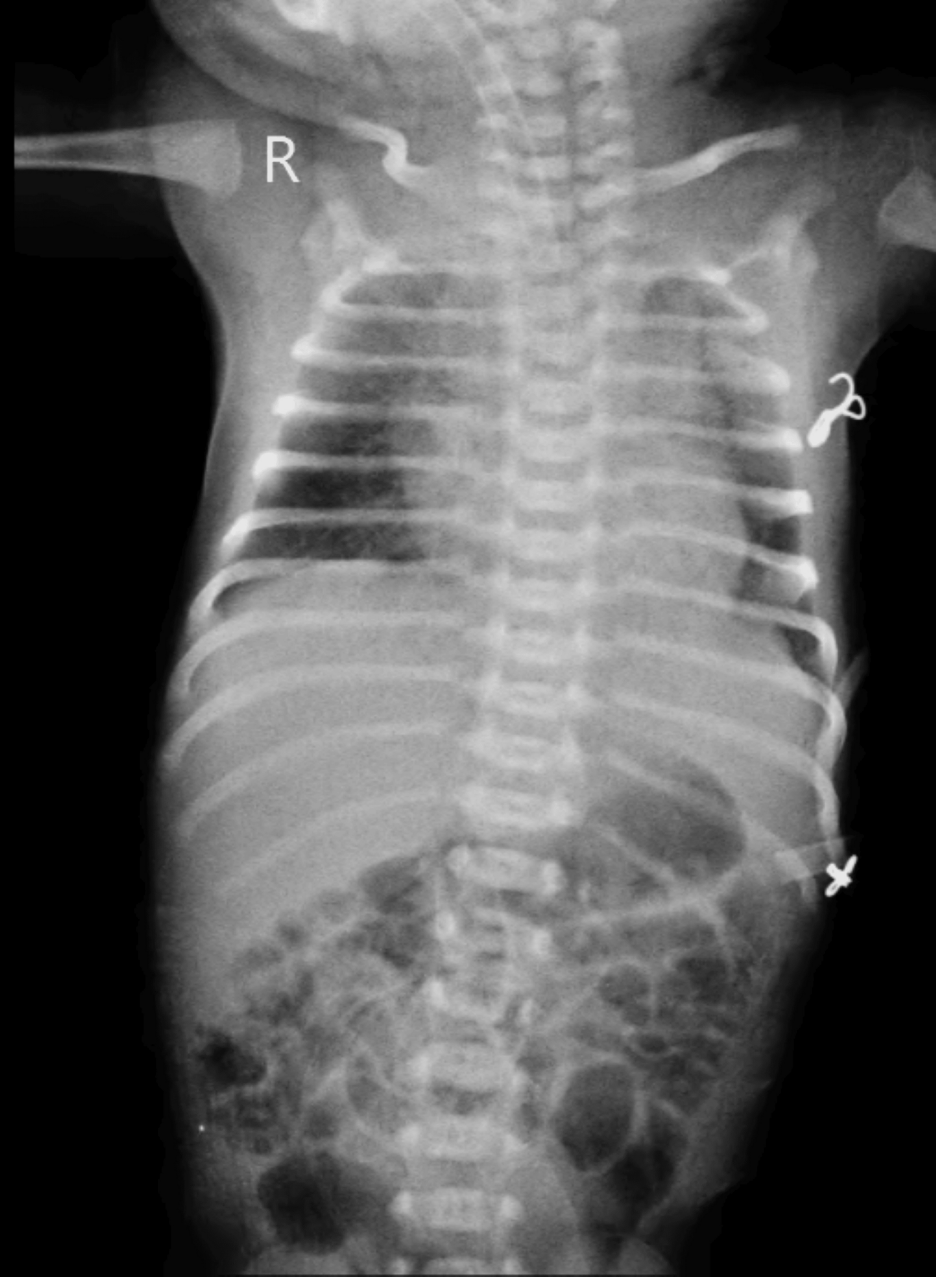
Bronchovesicular markings are present in the right middle zones and left upper, middle, and lower zones. There is a slight decrease in the costophrenic angle.

The chest X-ray shows prominent bronchovesicular markings in the right and left middle lung zones. These findings suggest increased visibility of the bronchial and vascular structures, which could be associated with conditions such as bronchitis, early interstitial lung disease, or simply a variant of normal, depending on the clinical context. The presence of these markings should be evaluated along with the patient’s symptoms and clinical findings to determine their significance shown in Figure 2.

Physiotherapy intervention

After obtaining the referral and consent the physiotherapy was started. The infant had physical therapy rehabilitation daily two times for 30 minutes each day. The protocol to be followed based on a rehabilitation timeframe is presented in Table 2. Goals with intervention and dosage are explained. In the below intervention, we explain how thoracic squeezing and percussion vibration with positioning help to improve the condition of infant in Table 2.

**Table 2:** Physiotherapy intervention with dosage

Sr No.	Goals	Intervention	Dosage
1	To reduce secretions	To create vibrations in the lung airways, chest percussion was applied by striking the chest with percussion cups, which resemble cups with suction. By releasing mucus, these vibrations aid in increased cough ejection.	5 repetitions* 2 sets every 3 hourly.
2.	To facilitate and profuse airway secretions	Thoracic squeezing technique is given as it stimulates the normal cough mechanism through the elevation of intrathoracic pressure.	Done for at least 10 minutes on each side.
3.	Improve ventilation and reduce work of breathing	Expiratory flow technique is given as it facilitates better airflow during expiration and helps in clearing the secretions.	2-3 repetitions for 5-10 minutes for 3 times a day.
4.	Postural drainage	Throughout the anterior segment draining of the left and right upper lobes, the infant was maintained in a flat, supine posture. The newborn was positioned such that they were 90 degrees forward during drainage. Next, the right and left lateral basal segments of the lower lobes were the first targets by using percussion over the topmost regions of the lower ribs. When applying percussion over the side of the chest, under the clavicles, and toward the lower abdomen region, care was taken to avoid putting direct pressure on the sternum.	Every 3 hours a day, 4-5 minutes.
5.	Suctioning	The objective of nasotracheal suctioning (NTS) during tracheal aspiration was to clear the nasopharyngeal and trachea of accumulated sputum, blood, lung secretions, liquid saliva, and other foreign objects. Throughout the procedure, the nurse removed a sterile, flexible, multi-eyed catheter using sub-atmospheric pressure. For the removal of the secretions, raise the infant's sub-atmospheric pressure to 80–100 mm Hg. Active moderate vibrations were delivered initially, followed by suction.	According to the severity of secretions.

Outcome measures

An outcome measure is a tool used to assess a patient’s current status. In Table 3, outcome measures are explained. Pre and post-assessment was taken. Thus, seven outcomes are taken which include, the APGAR score, oral motor assessment scale, Ballard score, length of hospital stay, Silverman Andersen Respiratory Severity Score, and arterial blood gas analysis.

**Table 3:** Outcome measures with pre- and post-assessment APGAR: appearance, pulse, grimace, activity, respiration; RSS: respiratory severity score

Sr. No	Outcome Measure	Pre-assessment	Post-assessment (Day 8)
1	APGAR score	6/10 (5 minutes)	10/10
2	Oral motor assessment scale	8/36	30/36
3	Ballard Score-(Neuromuscular maturity sign)	9/30	20/30
4	Ballard Score-(Physical maturity sign)	12/35	28/35
5	Length of hospital stay	28 days	
6	Silverman Andersen Respiratory Severity Score	7 (Impending respiratory failure)	4 (Impending respiratory failure)
7	Arterial Blood Gas analysis	Respiratory metabolic alkalosis	Normal

## Discussion

In this case, the thoracic squeezing technique and expiratory flow increase technique with chest physiotherapy were given in neonates with NRDS, given twice a day, showed results in which there was reduce in secretions. Also, there was an improvement in the breathing pattern of the baby. The outcomes were taken, such as APGAR score, oral motor assessment scale, Ballard score, length of hospital stay, Silverman Andersen Respiratory Severity Score, and arterial blood gas. Pre-assessment and post-assessment were drawn with skillful results.

The immaturity of the infant lung is the direct cause of hyaline membrane disease (HMD) and NRDS. Crucial variables include the immaturity of the lung’s shape, the chest wall’s higher compliance, the premature pulmonary lymphatics’ inefficiency, and the impaired production or function of surfactant [4]. Premature babies or those with other medical characteristics that put them at high risk for the condition at birth should be treated by a medical team experienced in managing respiratory difficulties in neonates [12]. Many preterm babies still struggle with oral feeding, even though improvements in neonatal critical care have led to a dramatic decline in the mortality rate of these babies. The supply of oral, tactile, kinaesthetic, vestibular, and auditory sensory inputs that are developmentally appropriate is defined as a means of preventing or minimizing adverse environmental impacts and promoting the development of preexisting fundamental abilities. Mouth sensorimotor therapies have been extensively studied because they address the oral tissues involved in feeding directly and specifically [13]. Oral feeding in the baby stage necessitates exact timing of breathing, swallowing, and sucking [14]. The emergence of oral feeding in preterm neonates may present difficulties because of prolonged hospital stays, respiratory problems, and other medical factors related to premature delivery [15].

For premature infants, chest physical therapy is safe. Suctioning, on the other hand, considerably modifies cardio-respiratory characteristics within the typical physiological range. As a result, chest physical therapy should only be used when required and in conjunction with continuous monitoring [16]. Changes in temperature, light, noise levels, and procedures for assessment and treatment are all experienced by the infant in the NICU. Sometimes the baby will be handled excessively, have uncomfortable procedures, be given pharmaceuticals, or be exposed to unpleasant stimuli.

The infant’s health could be in jeopardy due to the high likelihood of infection [17]. The technique of clearing the airways known as chest physiotherapy, or CPT entails the carer manually hammering the patient’s chest wall and positioning them in a way that permits mucus to exit. Little children undergoing physical therapy should ideally be put on their mother’s or the physiotherapist’s lap, with cushions to support them while they lie in the correct position [18]. When it comes to neuromuscular physiotherapy, physiotherapists concentrate on posture, parent education, passive range of motion exercises, and therapeutic handling [19]. One significant disadvantage is the fragile state in which these infants are born, which makes it challenging to get intensive therapy without running the risk of adding to the stress or harm. Moreover, variations in each newborn’s reaction to rehabilitation therapies could make it more difficult to standardize procedures and the need for extremely personalized care plans. It can be challenging to draw firm conclusions regarding the effectiveness of specific treatments due to this variation, which may also have an impact on the evaluation of results. For newborns with RDS, swift recovery is essential despite these difficulties.

In this case report, physiotherapy actions improve respiratory health, support healthy growth, and lessen the impact of respiratory problems, providing a holistic approach to newborn care. Early intervention in respiratory and neuromuscular function may improve both the short- and long-term results for newborns with NRDS, including fewer respiratory difficulties, shorter hospital stays, and better neurodevelopmental outcomes.

## Conclusions

This case study offers a physical rehabilitation strategy that enhances feeding, stability, respiratory function, and airway clearing in newborns with RDS. Thoracic squeezing, chest physiotherapy, and expiratory end flow technique help in reducing the secretions. Early physiotherapy helps improve outcomes in neonates with greater possibility. In summary, the case study underscores the potential benefits of physical rehabilitation as an adjunctive intervention for neonates diagnosed with RDS.
